# Structural and functional characterization of chitinase from carnivorous plant *Drosera adelae*

**DOI:** 10.1002/2211-5463.70110

**Published:** 2025-08-28

**Authors:** Kazunari Yoneda, Yuki Naruse, Yusaku Suizu, Tomohiro Araki, Yoshikazu Hoshi, Haruhiko Sakuraba, Junji Hayashi, Toshihisa Ohshima

**Affiliations:** ^1^ Department of Food and Life Sciences, School of Agriculture Tokai University Kumamoto Japan; ^2^ Graduate School of Agriculture Tokai University Kumamoto Japan; ^3^ Department of Applied Biological Science, Faculty of Agriculture Kagawa University Miki‐cho Japan; ^4^ Division of Bioscience and Bioindustry, Graduate School of Technology, Industrial and Social Sciences Tokushima University Japan; ^5^ Department of Biomedical Engineering, Faculty of Engineering Osaka Institute of Technology Japan

**Keywords:** carnivorous sundew plant, chitinase, crystal structure, *Drosera adelae*, GlcNAc interaction

## Abstract

A class I chitinase from the carnivorous sundew plant *Drosera adelae* was successfully expressed in the methylotrophic yeast *Pichia pastoris* and efficiently purified using a chitin affinity column. Enzymatic activity assays revealed that the enzyme showed a specific activity of 235.3 ± 10.2 U·mg^−1^. Crystallization of wild‐type and E167Q catalytic mutant chitinases yielded needle‐like microcrystals. X‐ray diffraction experiments were performed, and high‐resolution datasets were obtained at 1.73 Å and 1.57 Å, respectively. Structural analysis of diffraction data revealed that only the catalytic domain could be resolved in both crystal forms. Using AutoDock Vina, we performed docking simulations of two (GlcNAc)_4_ molecules at eight subsites (+4 to −4) of the catalytic domain of *D. adelae* chitinase to investigate their binding energies and conformations. Further, the structure of the chitin‐binding domain (hevein domain), which could not be resolved by X‐ray crystallography, was predicted using alphafold2. Based on this model, the binding conformation and binding energy of (GlcNAc)_3_ were analyzed using similar methods. In *D. adelae* chitinase, a characteristic tyrosine cluster consisting of Tyr174, Tyr199, and Tyr201 formed a unique structural feature that enabled recognition of the (GlcNAc)_4_ substrate. The hevein domain structures further indicated that the tyrosine cluster (Tyr41, Tyr43, Tyr50) in *D. adelae* chitinase may be involved in hydrogen bonding and CH/π interactions with (GlcNAc)_3_.

## Graphical abstract

A class I chitinase from the carnivorous plant *Drosera adelae* was expressed and purified using a yeast system, revealing high enzymatic activity. Structural analyses of the catalytic and chitin‐binding domains identified key tyrosine residues involved in substrate binding, offering insights into the enzyme's adaptation for insect digestion.

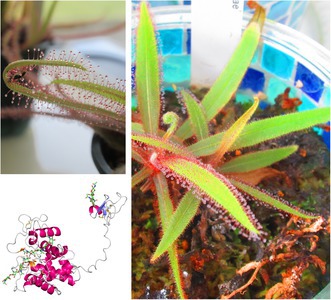

**Abbreviations**
BMGY: buffered glycerol complex medium
BMMY: buffered complex methanol medium
DTT: dithiothreitol
GlcNAc: *N*‐acetyl‐d‐glucosamine
PDB: Protein Data Bank
RMSD: root‐mean‐square deviation

Chitinase (EC3.2.1.14) is a glycoside hydrolase that catalyzes the hydrolysis of chitin, a polymer composed of β‐1,4‐linked *N*‐acetyl‐d‐glucosamine (GlcNAc) units. Chitinases play diverse physiological roles; in crustaceans and insects, which possess chitin in their bodies, chitinases are involved in molting and growth, whereas in fungi, they contribute to cell division. Chitinases also serve various functions in organisms without chitin; for example, in fungi parasitizing the shells of crustaceans, they degrade chitin, allowing its utilization from decaying matter, and in plants, they are implicated in defense against pathogenic infections [1]. In carnivorous plants, chitinases are involved in the digestion of insects. For example, in the carnivorous plant *Drosera adelae* (a species of sundew), chitinase is secreted from the glandular hairs on leaves to break down chitin in captured insects, thus aiding in nutrient absorption. Although chitinase typically functions as a ‘pathogen‐resistance enzyme’ that protects plants from bacterial infections, in carnivorous plants, it represents a rare evolutionary case in which the enzyme has been repurposed as an ‘insect‐digesting enzyme’ [2]. Consequently, many aspects of the functions and structures of chitinases from carnivorous plants remain poorly understood. Although the enzymatic functions of chitinases from the carnivorous plants *D. rotundifolia* and *D. capensis* have been reported, no crystal structures of these enzymes have been determined to date [3, 4, 5].

Chitinases are broadly classified into two groups: the glycosyl hydrolase 18 family (GH18) and 19 family (GH19) based on the structure of their catalytic domains. Plant chitinases are further divided into five major classes based on the similarities in their primary amino acid sequences [6]. Sequence analysis of *D. adelae* chitinase has revealed that it belongs to class I of the GH19 family. A distinguishing feature of class I chitinases is the presence of a chitin‐binding domain, also known as the hevein domain, at their N‐terminal region [6]. Proteomic analysis has revealed the presence of a class I chitinase belonging to the glycosyl hydrolase 19 family (GH19) in the digestive fluid of the carnivorous plant *D. adelae*, which plays a role in breaking down the exoskeletons of arthropods and releasing GlcNAc [2]. Based on these findings, the enzyme examined in this study has been considered to be primarily involved in insect digestion in *D. adelae*.

Chitinases with high enzymatic activities have significant potential for industrial applications [7]. For example, because many plant pathogens are fungi, chitinases are expected to be utilized as antimicrobial proteins that degrade fungal cell walls. Furthermore, their integration with organic agriculture offers potential applications as biopesticides in organic farming. They may also be used to control ectoparasites, such as fleas and ticks in livestock and pets. Further, chitinases are expected to contribute to postharvest technologies, including fungal control and pest management after crop harvest. In this study, we analyzed the function and three‐dimensional structure of chitinase from the carnivorous plant *D. adelae* in order to develop its novel application.

## Materials and methods

### Chemicals and culture media

Ethylene glycol chitin was obtained from FUJIFILM Wako Pure Chemicals Corp. (Osaka, Japan). Amino acid‐free Yeast Nitrogen Base (YNB) was purchased from Difco Laboratories (Detroit, MI, USA). G418 disulfate, dithiothreitol, iodoacetic acid, and cellulose powder were purchased from Nacalai Tesque (Kyoto, Japan). The oligonucleotide primers were obtained from FASMAC (Kanagawa, Japan). Calcofluor White stain and *Micrococcus luteus* were obtained from Sigma Aldrich (St. Louis, MO, USA). Chitin powder was purchased from Seikagaku Corporation (Kyoto, Japan). The protein marker for SDS/PAGE was purchased from Bio‐Helix (New Taipei, Taiwan). All other chemicals used were of analytical grade and commercially available. *N*‐Acetyl‐d‐glucosamine oligosaccharide (GlcNAc)_3–6_ was prepared as previously described [8].

YPD culture medium contained 2 g per 100 mL peptone, 1 g per 100 mL yeast extract, and 2 g per 100 mL glucose. The MD medium used to screen the histidine‐defective strains contained 1.34 g per 100 mL YNB, 40 μg per 100 mL biotin, 2 g per 100 mL glucose, and 1.8 g per 100 mL agar. The BMGY fermentation medium contained 2 g per 100 mL peptone, 1 g per 100 mL yeast extract, 1.34 g per 100 mL YNB, 40 μg per 100 mL biotin, 0.1 m potassium phosphate buffer (pH 6.0), 1.0% (v/v) glycerol, and 10 mg per 100 mL G418. The BMMY expression‐inducing medium contained 2 g per 100 mL peptone, 1 g per 100 mL yeast extract, 1.34 g per 100 mL YNB, 40 μg per 100 mL biotin, 0.1 m potassium phosphate buffer (pH 6.0), 1.0% (v/v) methanol, and 10 mg per 100 mL G418. All media were sterilized at 121 °C for 20 min.

### Cloning, expression, and purification

The gene encoding *D. adelae* chitinase (BAW35424.1; basic chitinase) was identified using the GenBank database and synthesized using a chemical method (FASMAC, Kanagawa, Japan). Gene synthesis of *D. adelae* chitinase was performed by excluding the signal peptide sequence (^1^MKISLLLLLF VVPLLSGTSA^20^). The gene ligated into the pUC19 vector contained a unique *Nde*I restriction site overlapping the 5′ initiation codon and a unique *Bam*HI restriction site proximal to the 3′ end of the termination codon. The synthesized 918‐bp fragment was digested with *Eco*RI and *Not*I, and ligated with the expression vector pPIC9K (Invitrogen, Waltham, MA, USA), which was previously linearized with *Eco*RI and *Not*I to generate pPIC9K‐chitinase. The QIAprep Spin Miniprep Kit (QIAGEN, Germantown, MD, USA) was used for plasmid extraction and purification. This was then used to transform the *Pichia pastoris* GS115 strain (Thermo Fisher Scientific, Cleveland, OH, USA).

The expression vector was linearized using *Sal*I restriction endonuclease, and the purified digestion products were integrated into *P. pastoris* GS115 competent cells using the lithium acetate transformation method. MD solid medium plates were used to screen positive clones into which genes were successfully integrated, and YPD solid medium plates containing 0.3–1.8 mg·mL^−1^ G418 were used to screen transformants with a high gene copy number.

### Expression of *D. adelae* chitinase in *P. pastoris*

*D. adelae* chitinase production was achieved by cultivation in a 500‐mL capacity flask (100 mL medium) at 30 °C with shaking at 2 ****g****. The seed solution was inoculated into 100 mL YPD medium until the OD_600nm_ reached 1.0. The cells were collected by centrifugation at 3500 ***g*** for 10 min.

Next, the cells were cultivated at 30 °C in 100 mL of BMGY medium until the optical density at 600 nm reached 1.0–2.0. The cells were collected after centrifugation at 3500 ***g*** for 10 min, suspended in 100 mL BMMY medium, and supplemented with 1.0% (v/v) methanol every 24 h to induce *D. adelae* chitinase expression. The induction process lasted 144–180 h at 30 °C and 2 ***g***. Cell suspensions fermented in shaker flasks were centrifuged using a low‐temperature high‐speed centrifuge at 3500 ***g*** for 10 min at 4 °C to obtain the fermentation supernatants.

The resulting supernatant was loaded onto a chitin column (30 × 60 mm; Seikagaku Corporation, Kyoto, Japan) equilibrated with 10 mm Tris–HCl buffer (pH 8.0) containing 0.5 m NaCl; the column was washed with equilibration buffer, and the enzyme was eluted in a stepwise manner using 0.1, 0.2, and 0.3 m acetic acid (20 mL each). The active enzyme fractions in the 0.3 m acetic acid effluents were pooled and dialyzed against 10 mm Tris–HCl buffer (pH 8.0) containing 0.2 m NaCl. The purity of *D. adelae* chitinase was assessed using SDS/PAGE (12.5% acrylamide, 1‐mm‐thick gels) and protein staining with Bullet CBB Stain One (Nacalai Tesque, Kyoto, Japan). Purification was performed at room temperature (approximately 25 °C).

### Analysis of (GlcNAc)_3–6_ digestion products

A 1 mm solution of (GlcNAc)_6_ was incubated with *D. adelae* chitinase at 40 °C for 30 min, and the reaction mixture was deproteinized using ultrafiltration (Amicon Ultra 3000 NMWL). The sample was then freeze‐dried, and the molecular masses of the enzymatic reaction products were analyzed using matrix‐assisted laser desorption/ionization time‐of‐flight mass spectrometry (MALDI‐TOF MS) (JEOL JMS‐S3000; Tokyo, Japan). For MALDI‐TOF MS analysis, the sample was first dissolved in 50 μL of water, and 1 μL of the solution was taken and diluted with 9 μL of water. As a matrix, 2,5‐dihydroxybenzoic acid (10 mg·mL^−1^) was used. Equal volumes (1 μL each) of the final sample solution and the matrix solution were spotted onto a mirror plate for measurement. Mass spectrometry analysis was performed in the positive detection mode. Further, TOF MS analysis was performed in the spiral mode.

Experiments using (GlcNAc)_3–5_ as the substrates were performed as follows: After reacting the enzyme solution with 1 mm (GlcNAc)_3–5_, the mixture was deproteinized and freeze‐dried. The samples were analyzed by liquid chromatography‐mass spectrometry (LC–MS) using an AccuTOF 4G LC‐plus system (JEOL). The freeze‐dried samples were dissolved in water before LC–MS measurement. Mass spectrometry analysis was performed in the positive detection mode.

### Molecular mass determination of recombinant *D. adelae* chitinase

The molecular mass of the chitinase was determined using MALDI‐TOF MS. Specifically, 0.9 mg of the purified chitinase was dissolved in a solution containing 50% acetonitrile, 0.05% formic acid, and 0.01% trifluoroacetic acid. Sinapinic acid (10 mg·mL^−1^) was used as the matrix. Equal volumes (1 μL each) of the final sample and matrix solutions were spotted onto a mirror‐finished plate for measurement. Mass spectrometric analysis was performed in the positive ion mode; TOF was operated in the linear mode.

### Site‐directed mutagenesis

Site‐directed mutagenesis was performed using inverse PCR with KOD FX DNA polymerase (Toyobo, Osaka, Japan). The pPIC9K‐chitinase (wild‐type) vector served as the template, and the following sets of oligonucleotide primers were used as mutagenic primers (mutations are underlined): 5′‐ATTGCTTTAAACAGCAGCAGGGTAATCCCG‐3′ and 5′‐CGGGATTACCCTGCTGCTGTTTAAAGCAAT‐3′ for E167Q mutant. The PCR protocol was as follows: 2 min at 94 °C for predenaturation, followed by 25 cycles of denaturation at 98 °C for 10 s and extension at 68 °C for 10 min. Wild‐type template DNA was digested with 20 U of *Dpn*I at 37 °C for 1 h. After transformation of *Escherichia coli* JM109 competent cells, plasmids were extracted and the DNA sequence was confirmed. The expression and purification processes used for the E167Q variant were similar to those of the wild‐type enzyme.

### Analysis of N‐terminal amino acid sequences

Before performing SDS/PAGE, chitinase was reduced and alkylated to break the disulfide bonds (SS bonds) using 30 mm DTT and 50 mm iodoacetic acid. The reduced and alkylated enzyme (about 10 μg) was subjected to SDS/PAGE as described above and then electroblotted onto a polyvinylidene difluoride membrane. The membrane was then stained with Coomassie Brilliant Blue R‐250 and destained. To determine the N‐terminal amino acid sequences, the protein band was subjected to automated Edman degradation using a PPSQ‐51A Protein Sequencer (Shimadzu, Kyoto, Japan).

### Chitinase assay and activity staining

Chitinase activity was assayed using a modified Schales procedure [9] with ethylene glycol chitin (water‐soluble chitin; model substrate) as the substrate (final concentration, 0.2%). A standard assay was performed at 40 °C in 50 mm sodium acetate buffer (pH 5.5) for 4 min. The reaction was terminated by cooling the samples in an ice‐cold bath, and the amount of reducing sugar generated was measured. One unit of chitinase activity was defined as the amount of enzyme that produced 1 μmol of reducing sugar per min. All experiments were independently performed in triplicate. A native‐PAGE gel containing 0.05% ethylene glycol chitin was prepared, and purified chitinase was subjected to electrophoresis. Chitinase activity was visualized using 0.01% Calcofluor White under UV illumination.

### Effect of temperature and pH on *D. adelae* chitinase activity

The effect of temperature on enzyme stability was determined by incubating the enzyme for 10 min at different temperatures in 10 mm Tris–HCl buffer (pH 8.0), and residual activity was measured using a standard assay for monitoring ethylene glycol chitin hydrolysis. To examine how pH affects enzyme stability, the enzyme was incubated for 20 min in buffers of various pH (at 25 °C), and residual activity was measured; the buffers (100 mm) used in these assays were acetic acid (pH 4.0–4.5), citrate (pH 5.0–5.5), potassium phosphate (pH 6.0–7.5), Tris–HCl (pH 7.5–9.0), with the buffer pH adjusted at room temperature. The same buffers were used to determine the optimal pH for the enzymatic activity.

### Cellulose binding assay

The purified wild‐type *D. adelae* chitinase was loaded onto a cellulose column (15 × 120 mm) equilibrated with 10 mm Tris–HCl buffer (pH 8.0); the column was washed with the equilibration buffer, and the enzyme was eluted in a stepwise manner using 0.5 and 2.5 m NaCl, and 0.3 m acetic acid (10 mL each). The *D. adelae* chitinase fraction was confirmed using SDS/PAGE. The cellulose binding assay was performed at room temperature (approximately 25 °C). The hydrolytic activity of cellulose was assayed according to the method described previously [3].

### Lytic activity measurement

Lytic activity was evaluated using the lyophilized cell wall of *Micrococcus luteus* as the substrate. The assay was performed as described previously [10].

### Crystallization, data collection, phasing, and refinement

For crystallization of the wild‐type enzyme, purified *D. adelae* chitinase in 10 mm Tris–HCl buffer (pH 8.0) was concentrated to 32.4 mg·mL^−1^ by ultrafiltration (Amicon Ultra 30 K NMWL). Crystals were obtained using the sitting‐drop vapor diffusion method, in which 2 μL of protein solution containing 2.5 mm (GlcNAc)_3_ was mixed with an equal volume of mother liquor composed of 0.8 m K_2_HPO_4_, 0.8 m NaH_2_PO_4_, and 0.1 m 4‐(2‐hydroxyethyl) piperazine‐1‐ethanesulfonic acid (HEPES) buffer (pH 7.5). Crystals of E167Q mutant enzyme were grown in sitting drops, in which 1 μL of enzyme solution (4.2 mg·mL^−1^) containing 2.5 mm (GlcNAc)_3_ was mixed with an equal volume of mother liquor composed of 15% PEG 8000, 40% 2‐propanol, and 0.1 m imidazole pH 6.5. In all cases, sitting drops were equilibrated against 50–100 μL of the reservoir solution in Compact Clover Crystallization Plates (Rigaku, Tokyo, Japan) at 20 °C for 1 month.

*D. adelae* chitinase crystals were flash‐cooled in liquid nitrogen at 100 K. The crystals were cryoprotected using 30% (*v*/*v*) ethylene glycol (Hampton Research, Aliso Viejo, CA, USA). Diffraction data were collected using monochromatic radiation of wavelength 1.0000 Å and a Pilatus detector system on the BL‐5A beamline at the Photon Factory Synchrotron Radiation Facility, Tsukuba, Japan. Data were processed using fastxds software [11].

Phase calculations were performed using the molecular replacement method and the program molrep [12], and the structure of *D. adelae* chitinase predicted using alphafold2 [13] was used as the search model. Models were further constructed using the program coot [14] and then refined to 1.73 Å (wild‐type) resolution using refmac5 [15]. Acetate and GlcNAc molecules were clearly visible in both the σ_A_‐weighted 2*F*_o_ − *F*_c_ and *F*_o_ − *F*_c_ density maps and were included in the latter part of the refinement. Water molecules were incorporated using coot [14]. The model geometry was analyzed using molprobity [16]. The initial phases of the E167Q mutant enzyme structure were determined using molrep in the ccp4 program suite [12]; the structure of chain A from the wild‐type enzyme served as the search model. Models were further built using coot [14] and refined to a 1.57 Å resolution using refmac5 [15]. An acetate molecule was included in the refinement process. The data collection and refinement statistics are presented in Table 2.

### Molecular docking simulation

The binding energies were calculated using autodock vina [17]. It employs a genetic algorithm to search for the most energetically favorable pose of a flexible small molecule within either the rigid or flexible binding site of a protein. autodock vina generated up to 10 binding modes for each docking run. The hydrogen‐added model structure file of the wild‐type *D. adelae* chitinase catalytic domain (PDB ID: 9JTR), along with the chitin‐binding domain (hevein domain) structure predicted using alphafold2, and the PDBQT (Protein Data Bank, Partial Charge (Q), & Atom Type (T)) molecular structure files of (GlcNAc)_3_ and (GlcNAc)_4_ were prepared using Dock Prep in autodock Tools. The areas used for docking calculations were as follows: for subsites A to C of the hevein domain of chitinase, center *x* = 17.7285, center *y* = 39.2715, center *z* = 2.14993, size *x* = 17.8565, size *y* = 10.7138, and size *z* = 13.2795 Å; for subsites +1 to +4 of the catalytic domain of chitinase, center *x* = −27.515, center *y* = 29.1509, center *z* = 12.5785, size *x* = 17.7921, size *y* = 18.0677, and size *z* = 14.9008 Å; and for subsites −1 to −4 of the catalytic domain of chitinase, center *x* = −29.2252, center *y* = 11.306, center *z* = 2.5639, size *x* = 12.2667, size *y* = 18.4801, and size *z* = 17.8861 Å. The identification of the subsites in the hevein domain was based on the structural information of rice chitinase (PDB ID: 2DKV) [18], while the subsites in the catalytic domain were identified using the structural information of rye class II chitinase (PDB ID: 4J0L) [19].

### Structure analysis

The chitin‐binding domain (hevein domain; Gln21‐Ser63), which could not be determined experimentally, was predicted using alphafold2 (colabfold v1.5.5). Root‐mean‐square deviation (RMSD) values, electrostatic potentials, and molecular graphics were calculated and generated using pymol v2.4.0 (http://www.pymol.org/).

## Results

### Production of recombinant protein

**Fig. 1:**
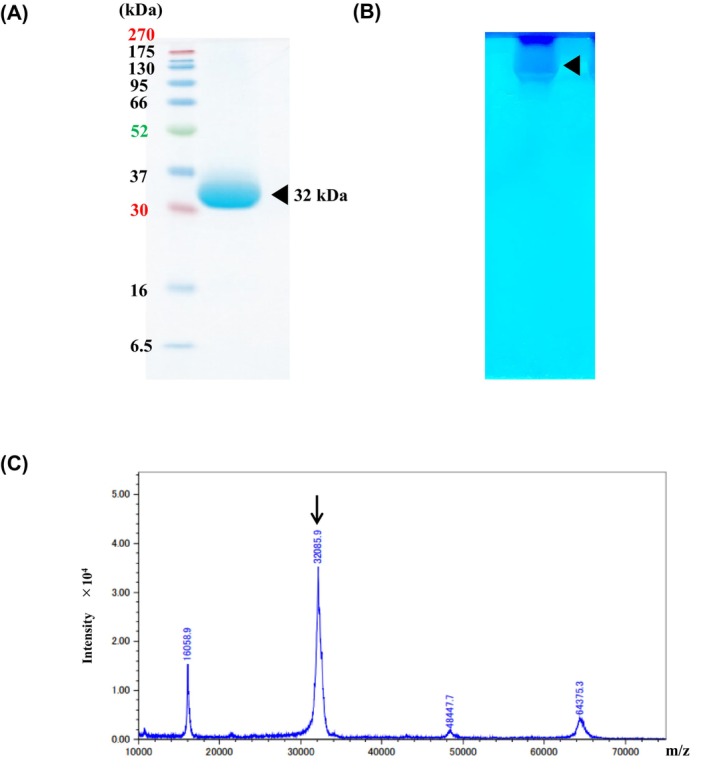
Purification and molecular mass determination of recombinant *D. adelae* chitinase. (A) Proteins separated by SDS/PAGE. Left lane, marker proteins (sizes shown on left); right lane, purified *D. adelae* chitinase. (B) Zymographic analysis of *D. adelae* chitinase activity. (C) MALDI‐TOF MS was used to determine the native molecular mass of the chitinase.

High chitinase activity was detected in the BMMY medium supernatant of recombinant *P. pastoris* cells, and a prominent band corresponding to the enzyme subunit was observed upon SDS/PAGE. The enzyme was readily purified from the BMMY medium supernatant in a single step of chitin column chromatography. From 100 mL of *P. pastoris* culture, approximately 45 mg of purified enzyme was obtained, which showed a specific activity of 235.3 ± 10.2 U·mg^−1^ (ethylene glycol chitin hydrolysis activity). The enzyme migrated as a single band on SDS/PAGE, and the molecular mass of the subunit was approximately 32 kDa (Fig. 1A). Furthermore, a chitinase activity band was detected by staining (Fig. 1B). However, the Glu167‐to‐Gln (E167Q) mutant completely lost its enzymatic activity (Table 1). The molecular mass of the enzyme was determined using MALDI‐TOF MS to be 32.0859 kDa (Fig. 1C).

**Table 1:** Biochemical characterization of *D. adelae* chitinase. n.d., no detectable activity.

Enzymatic characteristics of chitinase	*D. adelae* (This study)	*D. Rotundifolia* [3]	*O. sativa* (rice) [18, 20, 21]
Classification	Class I	Class I	Class I
Molecular mass (kDa)	32	33	32
Disulfide bonds (free Cys residues)	7, (3)	7, (3)	8, (1)
Number of Tyr residues, (mol%)	19, (5.65)	17, (5.01)	11, (3.41)
Amino acid sequence identity (%)	–	87	68
PDB codes	9JTR, 9JTP	–	2DKV
Quaternary structure, RMSD (Å)	Monomer, –	–, –	Monomer, 0.344
Catalytically important amino acid residues	Glu145, Glu167	Glu149, Glu171	Glu154, Glu176
Optimum pH	5.0–7.0	5.0–7.0	–
Optimum temperature (°C)	40	38	–
pH stability	4.0–8.0	–	–
Thermal stability (°C)	40	–	–
Substrate specificity			
Ethylene glycol chitin (U·mg^−1^), (U·μmol^−1^)	235.3, 3212.3	92, –	–, 253^a^
Mutant, activity (U·mg^−1^)	E167Q, n.d.	–	–
*Micrococcus luteus*	n.d.	–	–
Reaction products of (GlcNAc)_6_	(GlcNAc)_2,3,4_	–	(GlcNAc)_2,3,4_
Substrate cleavage specificity	Endo‐type	Endo‐type	Endo‐type
Activity and binding affinity toward cellulose	n.d., n.d.	n.d., –	–, –

^a^ The specific activity of *O. sativa* chitinase was reported in U·μmol^−1^.

The N‐terminal sequence of *D. adelae* chitinase was determined to be YVEFQ^21^QCGYQAGGALC^32^, which corresponds to the predicted sequence of *D. adelae* chitinase. The residual signal peptide fragment derived from the pPIC9K vector is underlined.

### Enzymological characteristics

The temperature dependency on the hydrolysis activity of ethylene glycol chitin between 20 °C and 70 °C was measured; the maximum activity was observed at approximately 40 °C, and the enzyme activity was not lost up to 40 °C. At 50 °C and 60 °C, the enzyme retained only 50% of its activity, while it was completely inactivated at 70 °C. The effect of pH on the enzyme activity was measured, and more than 80% of maximum activity was observed in the broad range of pH 5.0–7.0; the highest activity was around pH 5.5 (Table 1). The enzyme was stable over a wide range of pH values; the total activity was maintained between pH 4.0 and 8.0 after incubation at 30 °C for 20 min (Table 1).

A cellulose‐binding assay was performed using a cellulose column. *D. adelae* chitinase was detected in the flow‐through fraction, indicating that it did not bind to the cellulose column. These results demonstrate that chitinase does not possess cellulose‐binding ability (Fig. S1). No hydrolytic activity toward cellulose was detected (Table 1). Furthermore, lytic activity was evaluated using *Micrococcus luteus*, and the results revealed that *D. adelae* chitinase did not exhibit lytic activity (data not shown). Overall, *D. adelae* chitinase did not exhibit hydrolytic activity toward GlcNAc‐MurNAc (Table 1).

### Analysis of (GlcNAc)_3–6_ hydrolytic products

**Fig. 2:**
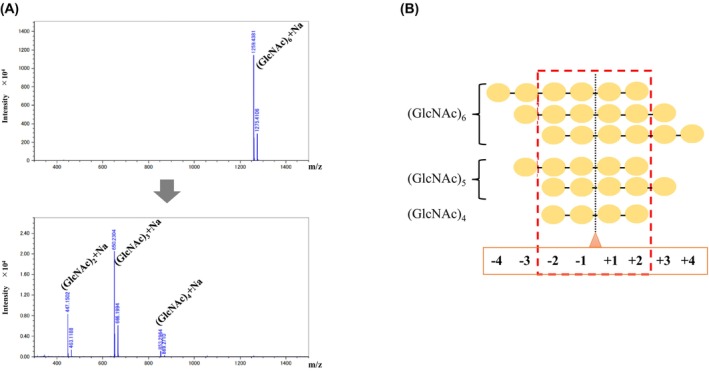
Analysis of (GlcNAc)_6_ digestion products. (A) The mass spectrum of (GlcNAc)_6_ digestion products produced by incubation with *D. adelae* chitinase. The top panel presents the measurement without chitinase, and the bottom panel shows the measurement after chitinase addition. (B) Binding modes of chitin oligosaccharides to *D. adelae* chitinase. Cleavage sites of (GlcNAc)_4–6_ targeted by the catalytic residues of *D. adelae* chitinase are indicated by orange triangles and black dashed lines. The numbers from −4 to +4 represent the subsites of chitinase. The essential subsites for substrate binding are highlighted using red dashed lines.

The (GlcNAc)_6_ degrading activity of *D. adelae* chitinase was analyzed using MALDI‐TOF MS. The enzyme hydrolyzed (GlcNAc)_6_ to yield two molecules of (GlcNAc)_3_ as the final product (Fig. 2A). Additionally, cleavage of (GlcNAc)_6_ into (GlcNAc)_2_ and (GlcNAc)_4_ was detected, followed by a predicted secondary reaction in which the enzymatic product (GlcNAc)_4_ was further hydrolyzed into two molecules of (GlcNAc)_2_ (Fig. 2A). To verify this, the hydrolysis products of (GlcNAc)_3–5_ were analyzed using chitinase. When (GlcNAc)_5_ was used as the substrate, (GlcNAc)_2_ and (GlcNAc)_3_ were generated as enzymatic products, whereas when (GlcNAc)_4_ was used, two molecules of (GlcNAc)_2_ were produced (Fig. S2A,B). No enzymatic activity was observed for (GlcNAc)_3_ (Fig. S2C).

### Overall structure

**Fig. 3:**
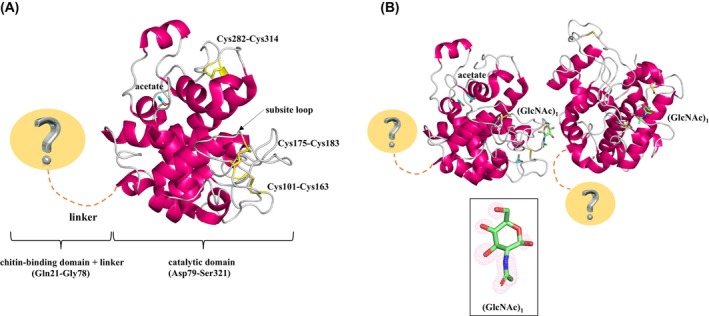
Overall structure of *D. adelae* chitinase. The structure of the E167Q mutant is shown in (A), and that of the wild‐type enzyme is shown in (B). Ribbon representation of the *D. adelae* chitinase monomer is shown. Catalytic domains are shown in red and white. The region involved in the chitin‐binding domain (hevein domain) and linker are indicated. Alpha‐helices are shown in red, and loop regions are shown in white. Cysteine residues involved in disulfide bonds (SS bonds) are shown as yellow sticks. GlcNAc (green) and acetate (cyan) molecules are shown as a stick model; red and blue indicate oxygen and nitrogen atoms, respectively. The final σ_A_‐weighted *F*_o_ − *F*_c_ omit electron density map of the GlcNAc molecule is shown at 3σ level.

We determined the structures of both wild‐type *D. adelae* chitinase bound to (GlcNAc)_1_/acetate (a fragment of the substrate and purification buffer) and the E167Q mutant enzyme bound to acetate using the molecular replacement method and then refined the structures (Table 2 and Fig. 3). The crystallographic *R*‐factor and free *R*‐factor of the structures of the (GlcNAc)_1_/acetate‐bound wild‐type enzyme and the acetate‐bound E167Q mutant were 0.208/0.238 and 0.202/0.234, respectively (Table 2). For the wild‐type *D. adelae* chitinase, two monomers were present in the asymmetric unit, with a solvent content of 47.61% and a Matthews coefficient of 2.35 Å^3^·Da^−1^. In the E167Q mutant, one monomer was present in the asymmetric unit, with a solvent content of 21.28% and a Matthews coefficient of 1.56 Å^3^·Da^−1^.

**Table 2:** X‐ray diffraction data collection and refinement statistics.

Statistics	Wild‐type enzyme	E167Q mutant
PDB codes	9JTR	9JTP
Data collection		
Synchrotron light source	Photon factory (KEK‐PF)	Photon factory (KEK‐PF)
Beam line	BL‐5A	BL‐5A
No. of frames	720	720
Oscillation width (°)	0.25	0.25
Detector distance (mm)	335.6	335.6
Wavelength (Å)	1.0000	1.0000
Exposure per frame (s)	0.5	0.5
Temperature (K)	100	100
Indexing and scaling		
Unit cell parameters (Å, °)	*a* = 73.222, *b* = 73.222, *c* = 192.998 α = β = 90, γ = 120	*a* = 43.120, *b* = 63.247, *c* = 72.894 α = β = γ = 90
Space group	*P*3_1_21	*P*2_1_2_1_2_1_
Resolution limit (Å)^a^	48.25–1.73 (1.76–1.73)	47.77–1.57 (1.60–1.57)
No. of unique reflections^a^	63 657 (3470)	26 673 (833)
Multiplicity^a^	9.5 (8.1)	5.9 (3.5)
⟨*I/*σ(*I*)⟩^a^	26.5 (2.2)	33.7 (2.7)
*R*_merge_ ^a^, ^b^	0.052 (0.787)	0.029 (0.404)
*R*_p.i.m._ ^a^, ^c^	0.018 (0.292)	0.013 (0.237)
Completeness (%)^a^	100.0 (100.0)	93.7 (60.4)
No. of chains per asymmetric unit	2	1
Overall B‐factor (Wilson plot, Å^2^)	26.1	21.0
Refinement		
Resolution range (Å)	38.43–1.73	47.77–1.57
*R*/*R*_free_ ^d^	0.208/0.238	0.202/0.234
No. of protein atoms	3714	1857
No. of water molecules	537	166
No. of ligands	Acetate, 4 (GlcNAc)_1_,_2_	Acetate, 1
RMSD		
Bond lengths (Å)	0.013	0.012
Bond angles (°)	1.659	1.648
Average B‐factors (Å^2^)	30.0	24.0
Ramachandran quality^d^		
Favored regions (%)	98.55	97.10
Outliers (%)	0.21	0

^a^ Values in parentheses are for the last resolution shell

^b^ *R*_merge_ = ∑_*h*_∑_*i*_|*I*_*i*_ (*h*) − <*I*(*h*)>|/∑_*h*_∑_*i*_|*I*_*i*_(*h*)|, where I_i_*i*_(*h*) is the intensity measurement for a reflection *h* and <*I* (*h*)> is the mean intensity for this reflection

^c^ *R*_p.i.m._ = ∑_*hkl*_ ({1/[*n*_hkl_ − 1]})^1/2^ ∑_*i*_ |*I*_*i*_ (*hkl*) − <*I*(*hkl*)>|/∑_*hkl*_ ∑_*i*_ *I*_*i*_(*hkl*)

^d^ *R*_free_ calculated with randomly selected reflections (4.9%).

The model of the acetate‐bound E167Q mutant (one monomer) contained 243 amino acid residues, one acetate, and 166 solvent water molecules (Fig. 3A). The model of the (GlcNAc)_1_/acetate‐bound wild‐type chitinase (two monomers) contained 486 amino acid residues, two (GlcNAc)_1_, four acetate, and 537 solvent water molecules (Table 2 and Fig. 3B). Although the Tyr199 residue in subunit B of the wild‐type enzyme appeared to be a slight outlier in the Ramachandran plot, inspection of the electron density map confirmed that the residue fit perfectly and was structurally consistent with the corresponding Tyr199 in subunit A (Fig. S3 and Table 2).

**Fig. 4:**
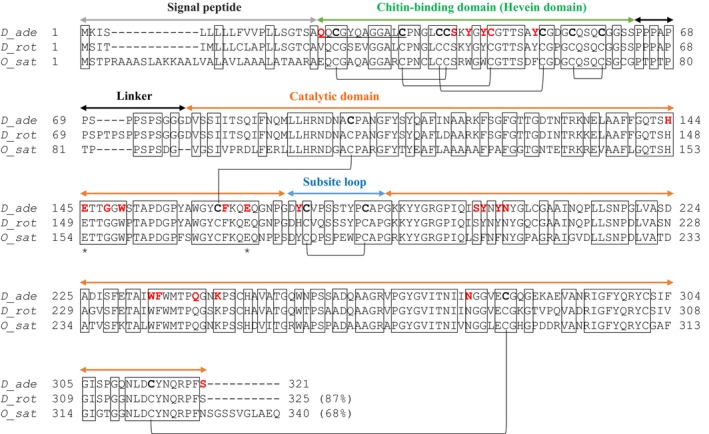
Amino acid sequence alignment of *D. adelae* (D_ade), *D. rotundifolia* (D_rot; AMM76171.1) [4] and *O. sativa* (O_sat; NP_001407416.1) [18] chitinase homologs using clustal w [22]. Disulfide bonds (SS bonds) are indicated as black bridges. Conserved residues are shown in the box; asterisks indicate the catalytic residues Glu145 and Glu167; red indicates the amino acid residues predicted to be involved in substrate binding. The regions corresponding to the signal peptide, hevein domain, linker, and catalytic domain are indicated by arrows. Further, the subsite loop region, which is reported to be important for subsite formation in *O. sativa*, is indicated by a blue arrow. The amino acid residues identified by N‐terminal amino acid sequencing are underlined.

Notably, for both wild‐type and E167Q mutant chitinases, clear electron density was only observed for the catalytic domain (Asp79‐Ser321), allowing model building in this region. In contrast, no electron density was observed in the chitin‐binding domain (hevein domain; Gln21‐Gly78); therefore, a structural model could not be constructed for this region (Fig. 3). The catalytic domain structure consists entirely of alpha helices. The three disulfide bonds within the catalytic domain (Cys101‐Cys163, Cys175‐Cys183, and Cys282‐Cys314) showed clear electron densities in both the wild‐type and E167Q mutant, allowing accurate model building. The free cysteine residues were identified as Cys206, Cys246, and Cys301 (Fig. 4). In the electron density map of (GlcNAc)_1_ shown in Fig. 3B, which was obtained through structural analysis, a clear electron density was observed for both the hydroxyl and *N*‐acetyl groups. However, no electron density corresponding to the added (GlcNAc)_3_ was detected, making it impossible to assign (GlcNAc)_3_ to the structure. Further details are provided in the Discussion section.

### Structural insight into the substrate‐binding simulation

**Fig. 5:**
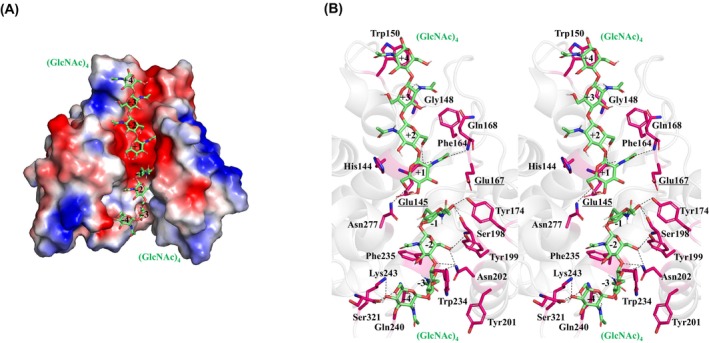
Proposed binding model of two (GlcNAc)_4_ molecules docked onto the catalytic domain structure of *D. adelae* chitinase determined in this study. (A) The molecular surface model color‐coded by electrostatic potential. (B) Wall‐eyed stereo view showing the detailed binding of (GlcNAc)_4_, with α‐helices in red, β‐sheets in blue, and loop regions in white. The two (GlcNAc)_4_ molecules are shown as green stick models, and the subsites are labeled from +4 to −4. Amino acid residues predicted to be involved in (GlcNAc)_4_ binding are labeled, and hydrogen bonds are indicated by black dashed lines. The oxygen and nitrogen atoms are shown in red and blue colors, respectively.

The docking of two molecules of (GlcNAc)_4_ into the catalytic site of *D. adelae* chitinase using autodock vina resulted in a calculated binding energy of −6.9 kcal·mol^−1^ at subsites +1 to +4, and −7.2 kcal·mol^−1^ at subsites −1 to −4 (Fig. 5A). In subsites +1 to +4, His144, Glu145, Gly148, Trp150, Phe164, Glu167, Gln168, and Asn277 were predicted to contribute toward the formation of a substrate‐binding pocket and its interaction with (GlcNAc)_4_ (Fig. 5B). At subsite +1, the O6 of GlcNAc forms a hydrogen bond with the side chain of Asn277, while the *N*‐acetyl group forms hydrogen bonds with the backbone of Gln168 and the catalytic residue Glu145. Another catalytic residue, Glu167, was found to be involved in the formation of subsite +1. At subsite +2, the O6 atom of GlcNAc was hydrogen‐bonded to the main‐chain oxygen atom of Glu145, and the aromatic residues His144 and Phe164 contributed to substrate‐binding pocket formation. At subsite +3, the *N*‐acetyl group of GlcNAc was hydrogen bonded to the main‐chain oxygen atom of Gly148. Additionally, the (GlcNAc)_1_ molecule observed in the crystal structure of the wild‐type enzyme was located at subsite +3. Although the (GlcNAc)_1_ molecule identified by structural analysis did not perfectly match the simulated (GlcNAc)_4_ molecule, it was found to bind in a similar orientation. At subsite +4, Trp150 formed a hydrophobic pocket, facilitating the interaction with GlcNAc (Fig. 5B).

At subsites −1 to −4, Tyr174, Ser198, Tyr199, Tyr201, Asn202, Trp234, Phe235, Gln240, Lys243, and Ser321 were predicted to contribute to the formation of the substrate‐binding pocket and its interaction with (GlcNAc)_4_ (Fig. 5B). At subsite −1, the O1 atom of GlcNAc formed hydrogen bonds with the side chains of Tyr174 (subsite loop) and Ser198. At subsite −2, O6 of GlcNAc formed hydrogen bonds with the side chains of Tyr199 and Asn202, while the O4 of GlcNAc formed a hydrogen bond with the side chain of Asn202. The aromatic amino acid residue Phe235 contributed to the formation of the substrate‐binding pocket. At subsite −3, the O5 of GlcNAc was hydrogen‐bonded to the side chain of Asn202. The aromatic amino acid residues Trp234 and Tyr201 contributed to the formation of the GlcNAc‐binding pocket. At subsite −4, the *N*‐acetyl group and O4 of GlcNAc formed hydrogen bonds with the side chains of Gln240 and Lys243, respectively. Additionally, the carboxyl group of the C‐terminal residue Ser321 formed hydrogen bonds with both the O4 and O6 atoms of GlcNAc (Figs 4 and 5B).

**Fig. 6:**
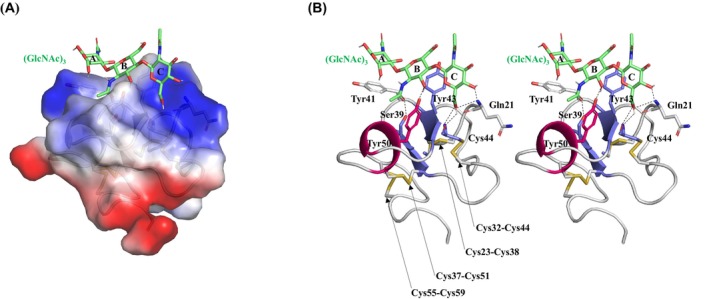
Predicted structure of the hevein domain of *D. adelae* chitinase and the (GlcNAc)_3_ binding simulation model. (A) Molecular surface model, color‐coded by electrostatic potential. (B) Wall‐eyed stereo view showing the binding of (GlcNAc)_3_, with α‐helices in red, β‐sheets in blue, and loop regions in white. (GlcNAc)_3_ molecules are shown as a green stick model; red and blue indicate oxygen and nitrogen atoms, respectively. For clarity, the three units of (GlcNAc)_3_ were labeled from A to C to indicate their positions. Disulfide bonds and amino acid residues involved in (GlcNAc)_3_ binding are represented as stick models. Hydrogen bonds are indicated by black dashed lines. *D. adelae* chitinase hevein domain structure was predicted using alphafold2 (colabfold v1.5.5).

Owing to the absence of an observable electron density for the chitin‐binding domain (hevein domain) in the crystal structure, its structure was predicted using alphafold2, and a docking simulation was performed with a (GlcNAc)_3_ molecule (Fig. 6A). Therefore, the binding energy between *D. adelae* chitinase and (GlcNAc)_3_ at positions A–C was calculated to be −4.8 kcal·mol^−1^. At the GlcNAc A–C positions, Gln21, Ser39, Tyr41, Tyr43, Cys44, and Tyr50 were predicted to contribute to the formation and interaction of the (GlcNAc)_3_ binding pocket (Fig. 6B). At position A, the aromatic residue Tyr41 formed a substrate‐binding pocket. At position B, the *N*‐acetyl group and O3 of GlcNAc formed hydrogen bonds with the side chains of Ser39 and Tyr50, respectively, while the aromatic residue Tyr43 contributed to pocket formation. At position C, the O4 and O6 atoms of GlcNAc formed hydrogen bonds with the main chains of Gln21 and Cys44, respectively (Fig. 6B).

A detailed structural comparison of the substrate‐binding sites between *D. adelae* and *O. sativa* chitinases, which share 68% amino acid sequence identity, is provided in the Discussion.

## Discussion

Based on the proteomic information on *D. adelae*, a class I chitinase was identified and its gene was synthesized. Initially, the gene was ligated into an *E. coli* expression vector, and large‐scale protein expression was attempted in *E. coli*. However, the chitinase gene product was barely detectable (data not shown). The enzyme contains a cysteine‐rich amino acid sequence with 17 cysteine residues and was predicted to possess seven disulfide bonds, suggesting that proper expression in the *E. coli* cytoplasm was inherently challenging because of the reducing environment that impairs disulfide bond formation (Fig. 4). Use of the yeast secretion expression system in *P. pastoris* successfully enabled the large‐scale purification of chitinase (Fig. 1A). Chitinase activity was confirmed by staining with native PAGE containing ethylene glycol chitin. A clear activity band was observed at the top of the separating gel, indicating that the enzyme did not efficiently migrate into the gel. This phenomenon was attributed to the strong interactions between the chitin‐binding domain (hevein domain) and the model substrate, ethylene glycol chitin, within the gel matrix (Fig. 1B). These results suggest that *D. adelae* chitinase possesses a chitin‐binding domain, which is a characteristic feature of class I chitinases. The chitinase activity was measured using the modified Schales method. The results revealed that the activity of *D. adelae* chitinase was approximately 12.7 times higher than that of *O. sativa* class I chitinase, a noncarnivorous plant enzyme whose function and three‐dimensional structure have already been determined (Table 1) [18, 20]. However, this specific activity value may vary due to differences between lots or batches of ethylene glycol chitin, such as partial deacetylation. The optimal pH for the enzyme was found to be broad, ranging from pH 5.0 to 7.0, similar to that of *D. rotundifolia* [3]. These findings suggest that the high activity and broad pH range of *D. adelae* chitinase may reflect physiological adaptations of the enzyme for digesting insects in its natural environment. Furthermore, in substrate specificity assays, *D. adelae* chitinase showed no reactivity toward cellulose or GlcNAc‐MurNAc. Mass spectrometry analysis of the cleavage pattern of (GlcNAc)_4–6_ revealed that the enzyme functioned as an endo‐type chitinase. This characteristic was consistent with that of other known chitinases (Table 1) [21]. Furthermore, based on the analysis of the enzymatic reaction products of (GlcNAc)_4–6_, the four subsites from +2 to −2 were determined to be essential for chitinase activity. Collectively, these findings strongly suggest that the region encompassing subsites +2 to −2 constitutes a critical interface for substrate recognition and binding (Fig. 2B).

We experimentally demonstrated that chitinase binds strongly to chitin columns, indicating that the enzyme has a high affinity for chitin. For the *D. adelae* chitinase, 0.3 m acetic acid was used for elution during purification on a chitin column. This was because the enzyme was so strongly bound to the column that it did not elute even with a 5 m NaCl elution buffer. These findings suggested that the enzyme interacted tightly with its chitin substrate. Such strong substrate interactions may also contribute to its high enzymatic activity. In the early phase of the study, the molecular mass of the native enzyme was determined by gel filtration chromatography using a TSKgel G3000SWXL column (7.8 × 300 mm, Tosoh, Tokyo, Japan). However, the elution time was significantly delayed, likely because of interactions between the chitin‐binding domain of chitinase and the matrix of the gel filtration column. The calculated molecular mass of the enzyme was unrealistically low (less than 0.5 kDa), and accurate measurements could not be achieved using this method (data not shown). Therefore, the molecular mass was determined using MALDI‐TOF MS, and the enzyme was clarified to function as a monomer (Fig. 1C).

A comparison of the amino acid sequences revealed that *D. adelae* chitinase was characterized by a greater number of tyrosine residues than those in the other chitinases (Table 1 and Fig. 4). As discussed below, structural comparisons demonstrated that in *D. adelae* chitinase, tyrosine residues formed clusters in both the hevein and catalytic domains. These tyrosine clusters were predicted to play a critical role in enzyme binding to its substrate, GlcNAc. Although structural simulation analyses have been reported for chitinases from the carnivorous plants *D. rotundifolia* and *D. capensis*, no X‐ray crystallographic data have been experimentally obtained to date [3, 5]. This was likely owing to the fact that class I chitinases are multidomain enzymes containing a hevein domain, which confers significant structural flexibility and makes crystallization extremely challenging. An amino acid sequence comparison demonstrated that *D. adelae* chitinase contained two catalytic residues, Glu145 and Gln167 (Fig. 4). Glu145 was predicted to act as a proton donor, whereas Gln167 was predicted to enhance the nucleophilicity of water molecules, as suggested by previous studies on related enzymes [23]. Based on the results of the functional analyses of *D. adelae* chitinase, the E167Q mutant enzyme was found to be completely inactive (Table 1). Therefore, the E167Q mutant enzyme was crystallized under the assumption that it would reduce domain flexibility. Reducing structural fluctuations was hypothesized to facilitate the structural analysis of enzyme‐substrate or enzyme–inhibitor complexes. Although crystallographic experiments were performed with this objective, the complete structure of the enzyme‐substrate complex could not be determined. Furthermore, despite crystallization of the enzyme in the presence of (GlcNAc)_3_, only (GlcNAc)_1_ was visible in the crystal structure. This was likely not because of the enzymatic degradation of (GlcNAc)_3_, but rather because of molecular flexibility, which resulted in electron density being observable only for (GlcNAc)_1_. In this study, the crystal structures of both the wild‐type and the E167Q mutant enzyme were determined, and the overall structures were almost identical (RMSD = 0.287 Å, 197 to 197 atoms). As the crystallization conditions for the wild‐type and E167Q mutant enzyme were completely different, as described in the Materials and methods section, the hevein domain structure was expected to be resolved in one of them. However, electron density was unfortunately observed only for the catalytic domain in both the wild‐type and E167Q mutant structures. The electron density of the hevein domain appears to be disordered due to interdomain flexibility; however, the possibility that degradation occurred during crystallization and prevented observation of the electron density cannot be completely ruled out.

**Fig. 7:**
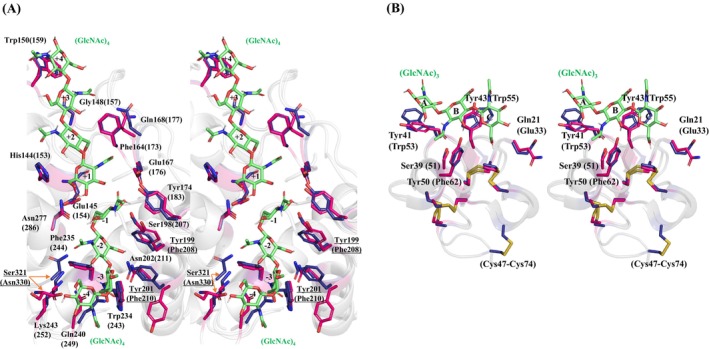
Wall‐eyed stereo views of the structural superimposition of the catalytic and hevein domains of *D. adelae* chitinase with *O. sativa* chitinase (PDB ID: 2DKV). (A) Superimposition of catalytic domains. (B) Superimposition of hevein domains. Parentheses indicate amino acid residue numbers of *O. sativa* chitinase. Amino acid residues predicted to be involved in (GlcNAc)_4_ binding are shown as red sticks for *D. adelae* chitinase and as navy blue sticks for *O. sativa* chitinase. Atom coloring is the same as that used in Fig. 5. *D. adelae* chitinase hevein domain structure was predicted using alphafold2 (colabfold v1.5.5).

Among the class I chitinases whose crystal structures have already been determined, the one with the highest amino acid sequence identity to *D. adelae* chitinase was *O. sativa* chitinase (PDB ID: 2DKV) (Fig. 4). By superimposing the (GlcNAc)_3–4_ complex structures of the catalytic and hevein domains of the *D. adelae* chitinase elucidated in this study and those of the *O. sativa* chitinase, we could clarify the structure–function relationship of chitinases. Structural superposition of the catalytic domains yielded an RMSD of 0.369 Å (209 to 209 atoms), while superposition of the predicted hevein domains resulted in an RMSD of 0.334 Å (34 to 34 atoms). A structural comparison of the catalytic domains indicated that most of the amino acid residues involved in (GlcNAc)_4_ binding were conserved between *D. adelae* and *O. sativa* chitinases. Among these, Tyr174, which is responsible for recognizing GlcNAc in the subsite loop region (corresponding to Tyr183 in *O. sativa* chitinase [18]), was highly conserved in the three‐dimensional structure, underscoring its critical role in substrate recognition (Figs 4 and 7A). Notably, Tyr199 and Tyr201 at subsites −1 and −3 in *D. adelae* chitinase were substituted with Phe208 and Phe210, respectively, in *O. sativa* chitinase. These substitutions resulted in the loss of hydroxyl groups, potentially affecting substrate interactions at these subsites. This structural analysis revealed that *D. adelae* chitinase forms a tyrosine cluster at subsites −1 to −3 (Fig. 7A). Furthermore, the C‐terminal Ser321 residue of *D. adelae* chitinase was found to be important for binding to GlcNAc at subsite −4. In *O. sativa* chitinase, this residue is substituted with Asn330, and the spatial positions of these residues do not overlap in the three‐dimensional structures, suggesting differences in GlcNAc binding affinity. Considering the structural differences observed at the C termini of the two enzymes, these variations may contribute to the differences in their enzymatic activities (Figs 4 and 7A).

Structural superposition of the hevein domains of *D. adelae* chitinase and *O. sativa* chitinase revealed that the hevein domain of *D. adelae* chitinase contains four disulfide bonds (Cys23‐Cys38, Cys32‐Cys44, Cys37‐Cys51, and Cys55‐Cys59), whereas the hevein domain of *O. sativa* chitinase contains an additional disulfide bond (Cys47‐Cys74), resulting in a total of five disulfide bonds (Figs 6B and 7B). Further, Tyr41, Tyr43, and Tyr50 in *D. adelae* chitinase were substituted with Trp53, Trp55, and Phe62, respectively, in *O. sativa* chitinase. Notably, as observed in the catalytic domain, *D. adelae* chitinase forms a tyrosine cluster in the hevein domain (Fig. 7B). These tyrosine residues were found to potentially interact with the GlcNAc via hydrogen bonding and CH/π interactions. Although previous *in silico* analyses of the three‐dimensional structure of *D. rotundifolia* chitinase suggested that tyrosine residues play a key role in CH/π interactions with the chitin substrate [3], the present study is the first to provide structural evidence supporting this interaction.

## Conflict of interest

The authors declare no conflict of interest.

## Author contributions

KY conceived and designed the study and wrote the manuscript. KY, YN, and YS performed the experiments and analyzed the data. KY, TA, YH, HS, JH, and TO contributed to manuscript preparation.

## Supporting information


**Fig. S1.** Cellulose binding assay.

**Fig. S2.** Analysis of (GlcNAc)_3–5_ digestion products.

**Fig. S3.** Ramachandran plot and electron density map of Tyr199 in *D. adelae* chitinase.

## Data Availability

Atomic coordinates and structural factors have been deposited in the Protein Data Bank (https://www.rcsb.org/; IDs: 9JTR and 9JTP).
