# Supplementary material for: Structural and functional characterization of chitinase from carnivorous plant Drosera adelae
Source: FEBS Open Bio. 2025 Aug 28;15(12):1930–44. doi: 10.1002/2211-5463.70110 (PMC12667207; doi:10.1002/2211-5463.70110)
Supplement: Supplementary file 1 — Fig. S1. Cellulose binding assay. [file FEB4-15-1930-s002.pdf]

Supplementary Figure 1

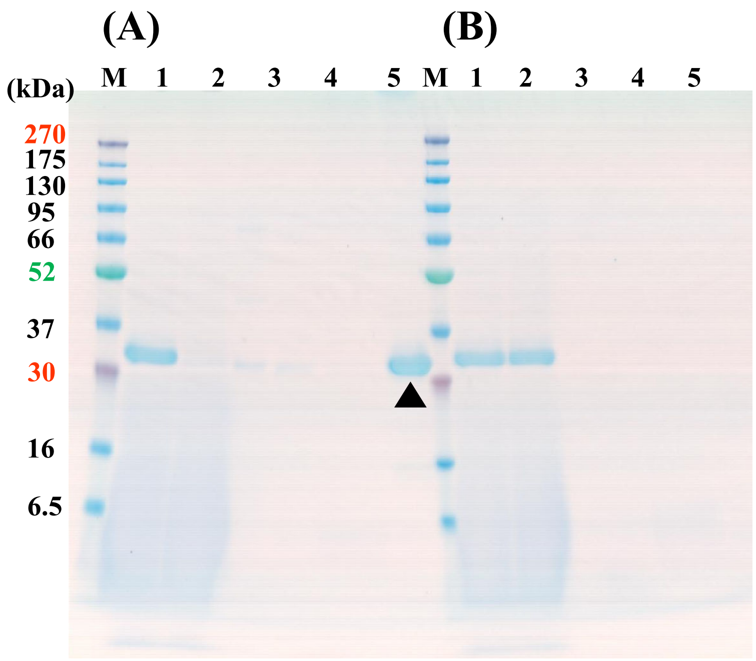

Supplementary Figure1. Cellulose binding assay. Binding experiments of *D. adelae* chitinase were performed using a chitin affinity column (A) and a cellulose column (B). Proteins separated by SDS-PAGE. Lane M, marker proteins (sizes shown on left); lane 1, BMMY medium supernatant before purification; lane 2, flow through fraction; lanes 3 and 4, 0.5 M and 2.5 M NaCl elution fractions, respectively; lane 5, 0.3 M acetic acid elution fraction.
