# Supplementary material for: Structural and functional characterization of chitinase from carnivorous plant Drosera adelae
Source: FEBS Open Bio. 2025 Aug 28;15(12):1930–44. doi: 10.1002/2211-5463.70110 (PMC12667207; doi:10.1002/2211-5463.70110)
Supplement: Supplementary file 2 — Fig. S2. Analysis of (GlcNAc)3–5 digestion products. [file FEB4-15-1930-s001.pdf]

Supplementary Figure 2

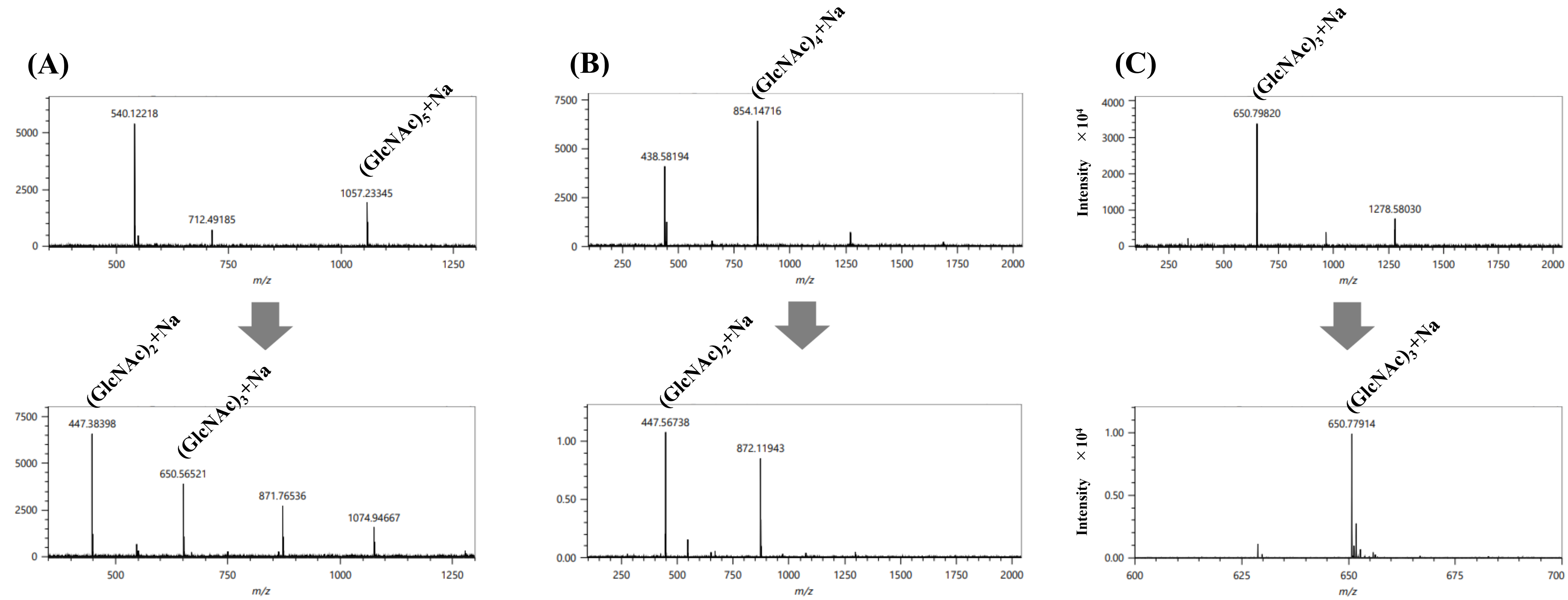

Supplementary Figure2. Analysis of $(GlcNAc)_{3\text{-}5}$ digestion products. (A) Mass spectrum of the digestion products of $(GlcNAc)_5$ following incubation with *D. adelae* chitinase at 40°C for 30 minutes. (B) Detection results of the enzymatic reaction products of $(GlcNAc)_4$, and (C) those of $(GlcNAc)_3$. The upper panels display the measurements without chitinase, whereas the lower panels show the results after chitinase addition.
