# Supplementary material for: Structural and functional characterization of chitinase from carnivorous plant Drosera adelae
Source: FEBS Open Bio. 2025 Aug 28;15(12):1930–44. doi: 10.1002/2211-5463.70110 (PMC12667207; doi:10.1002/2211-5463.70110)
Supplement: Supplementary file 3 — Fig. S3. Ramachandran plot and electron density map of Tyr199 in D. adelae chitinase. [file FEB4-15-1930-s003.pdf]

Supplementary Figure 3

(A)

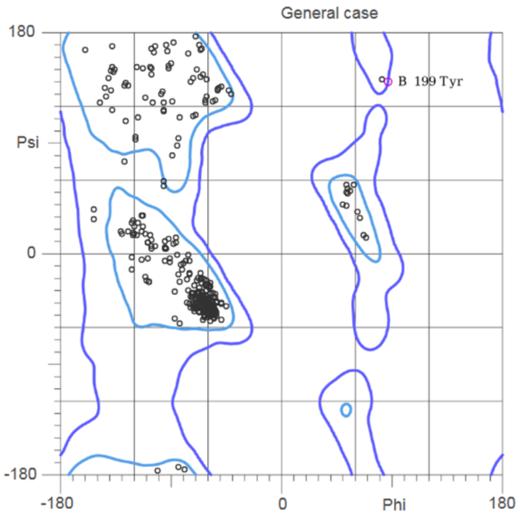

(B)

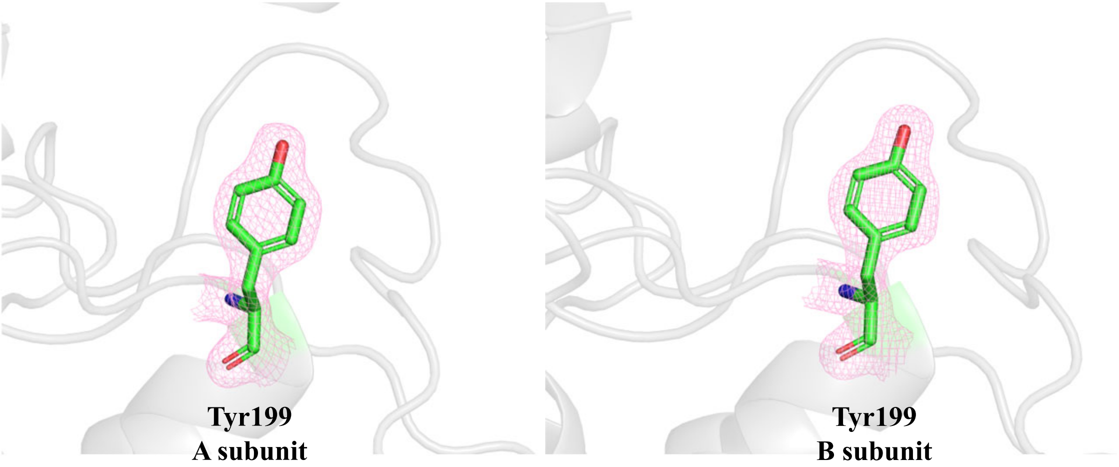

Supplementary Figure3. Ramachandran plot of the *D. adelae* chitinase wild-type (A), and the structures and electron density maps of Tyr199 in both subunits (B).
